# Coated or chewable aspirin and a hybrid air pollution mitigation strategy in patients with atherosclerotic disease: rationale and design of the COATED-AIR randomized trial

**DOI:** 10.1093/ehjcvp/pvag071

**Published:** 2026-09-23

**Authors:** Behnood Bikdeli, Azita H Talasaz, Hooman Bakhshandeh, Mohammad Sadegh Hassanvand, Hessam Kakavand, Sepehr Jamalkhani, Sina Rashedi, Sara Tayyebi, Mohammadreza Babaei, Erfan Kohansal, Hamed Ghoshouni, Yasmin Mohtasham Kia, Mahsa Nikkhoo, Bahram Mohebbi, Maryam Aghakouchakzadeh, Saeed Alipour-Parsa, Armin Elahifar, Ehsan Khalilipur, Razieh Omidvar, Maryam Mehrpooya, Seyed Hashem Sezavar, David Jimenez, Luis Jara Palomares, Saeideh Mazloomzadeh, Naser Hadavand, Mehdi Ansari Dogaheh, Majid Maleki, Feridoun Noohi, Luis Ortega-Paz, Jayant K Raikhelkar, Philippe Gabriel Steg, Renato D Lopes, Raffaele De Caterina, Mehdi H Shishehbor, Loren Laine, Dominick J Angiolillo, Sanjay Rajagopalan, Ajay J Kirtane, Gregory Piazza, Gregory Y H Lip, Roxana Mehran, Harlan M Krumholz, Paul M Ridker, Parham Sadeghipour

**Affiliations:** Division of Cardiology, Heart and Vascular Institute, Mass General Brigham, Brigham and Women’s Hospital, Boston, MA 02115, USA; Thrombosis Research Group, Brigham and Women’s Hospital, 75 Francis Street, Boston, MA 02115, USA; Harvard Medical School, Boston, MA 02115, USA; YNHH/Yale Center for Outcomes Research and Evaluation (CORE), New Haven, CT, USA; Department of Pharmacotherapy and Outcomes Sciences, Virginia Commonwealth University, Richmond, VA 23298, USA; Department of Pharmacy, New York-Presbyterian Hospital, Columbia University Medical Center, New York, NY 10032, USA; Vascular Disease and Thrombosis Research Center, Rajaie Cardiovascular Institute, Tehran 1995614331, Iran; Cardiovascular Epidemiology Research Center, Rajaie Cardiovascular Institute, Tehran 1995614331, Iran; Clinical Trial Center, Rajaie Cardiovascular Institute, Tehran 1995614331, Iran; Department of Environmental Health Engineering, School of Public Health, Tehran University of Medical Sciences, Tehran 1417613151, Iran; Center for Air Pollution Research (CAPR), Institute for Environmental Research (IER), Tehran University of Medical Sciences, Tehran 1417613151, Iran; Department of Clinical Pharmacy and Pharmacoeconomics, School of Pharmacy, Iran University of Medical Sciences, Tehran 1114133314, Iran; Vascular Disease and Thrombosis Research Center, Rajaie Cardiovascular Institute, Tehran 1995614331, Iran; Division of Cardiology, Heart and Vascular Institute, Mass General Brigham, Brigham and Women’s Hospital, Boston, MA 02115, USA; Thrombosis Research Group, Brigham and Women’s Hospital, 75 Francis Street, Boston, MA 02115, USA; Harvard Medical School, Boston, MA 02115, USA; Vascular Disease and Thrombosis Research Center, Rajaie Cardiovascular Institute, Tehran 1995614331, Iran; Vascular Disease and Thrombosis Research Center, Rajaie Cardiovascular Institute, Tehran 1995614331, Iran; Vascular Disease and Thrombosis Research Center, Rajaie Cardiovascular Institute, Tehran 1995614331, Iran; Vascular Disease and Thrombosis Research Center, Rajaie Cardiovascular Institute, Tehran 1995614331, Iran; Cardiovascular Epidemiology Research Center, Rajaie Cardiovascular Institute, Tehran 1995614331, Iran; Vascular Disease and Thrombosis Research Center, Rajaie Cardiovascular Institute, Tehran 1995614331, Iran; Vascular Disease and Thrombosis Research Center, Rajaie Cardiovascular Institute, Tehran 1995614331, Iran; Skin Research Center, Shahid Beheshti University of Medical Sciences, Tehran 1989934148, Iran; Vascular Disease and Thrombosis Research Center, Rajaie Cardiovascular Institute, Tehran 1995614331, Iran; Tehran Heart Center, Cardiovascular Diseases Research Institute, Tehran University of Medical Sciences, Tehran 1411713138, Iran; Cardiovascular Research Center, Shahid Modarres Hospital, Shahid Beheshti University of Medical Sciences, Tehran 1998734383, Iran; Cardiovascular Intervention Research Center, Rajaie Cardiovascular Institute, Tehran 1995614331, Iran; Cardiovascular Intervention Research Center, Rajaie Cardiovascular Institute, Tehran 1995614331, Iran; Vascular Disease and Thrombosis Research Center, Rajaie Cardiovascular Institute, Tehran 1995614331, Iran; Department of Cardiology, Imam Khomeini Hospital Complex, Tehran University of Medical Sciences, Tehran 1419733141, Iran; Research Center for Prevention of Cardiovascular Diseases, Institute of Endocrinology and Metabolism, Tehran 1411713119, Iran; Respiratory Department, Hospital Ramón y Cajal and Instituto Ramón y Cajal de Investigación Sanitaria IRYCIS, Madrid 28034, Spain; CIBER Enfermedades Respiratorias (CIBERES), Madrid 07110, Spain; Medicine Department, Universidad de Alcalá, Madrid 28801, Spain; Medical Surgical Unit of Respiratory Diseases, Virgen del Rocio Hospital and Instituto de Biomedicina, Seville 41013, Spain; Cardiovascular Epidemiology Research Center, Rajaie Cardiovascular Institute, Tehran 1995614331, Iran; Vascular Disease and Thrombosis Research Center, Rajaie Cardiovascular Institute, Tehran 1995614331, Iran; Drug and Food Control Department, Faculty of Pharmacy, Kerman University of Medical Sciences, Kerman 76169-13555, Iran; Vascular Disease and Thrombosis Research Center, Rajaie Cardiovascular Institute, Tehran 1995614331, Iran; Vascular Disease and Thrombosis Research Center, Rajaie Cardiovascular Institute, Tehran 1995614331, Iran; Division of Cardiology, University of Florida College of Medicine, Jacksonville, FL 32209, USA; Division of Cardiology, Department of Medicine, Columbia University Irving Medical Center, NY 10032, USA; French Alliance for Cardiovascular Trials, Paris 75018, France; Université Paris-Cité, Paris 75006, France; INSERM Unité 1148, Paris 75018, France; Assistance Publique–Hôpitaux de Paris (AP-HP), Hôpital Bichat, Paris 75018, France; Department of Cardiology, Duke University, Durham, NC 27708, USA; University Cardiology Division, Pisa University Hospital, Pisa 56126, Italy; University Hospitals, Harrington Heart and Vascular Institute, Case Western Reserve University School of Medicine, Cleveland, OH 44106, USA; Section of Digestive Diseases, Yale School of Medicine, New Haven, CT 06510, USA; VA Connecticut Healthcare System, West Haven, CT 06516, USA; Division of Cardiology, University of Florida College of Medicine, Jacksonville, FL 32209, USA; University Hospitals, Harrington Heart and Vascular Institute, Case Western Reserve University School of Medicine, Cleveland, OH 44106, USA; Division of Cardiology, University of Florida College of Medicine, Jacksonville, FL 32209, USA; Division of Cardiology, Department of Medicine, Columbia University Irving Medical Center, NY 10032, USA; Division of Cardiology, Heart and Vascular Institute, Mass General Brigham, Brigham and Women’s Hospital, Boston, MA 02115, USA; Thrombosis Research Group, Brigham and Women’s Hospital, 75 Francis Street, Boston, MA 02115, USA; Harvard Medical School, Boston, MA 02115, USA; Institute of Life Course and Medical Sciences, University of Liverpool, Liverpool L69 7ZX, United Kingdom; Department of Clinical Medicine, Aalborg University, Aalborg 9220, Denmark; Department of Cardiology, Lipidology and Internal Medicine with Intensive Coronary Care Unit, Medical University of Bialystok, Bialystok 15-089, Poland; Icahn School of Medicine at Mount Sinai, New York, NY 10029, USA; YNHH/Yale Center for Outcomes Research and Evaluation (CORE), New Haven, CT, USA; Section of Cardiovascular Medicine, Yale School of Medicine, New Haven, CT 06510, USA; Department of Health Policy and Management, Yale School of Public Health, New Haven, CT 06510, USA; Division of Cardiology, Heart and Vascular Institute, Mass General Brigham, Brigham and Women’s Hospital, Boston, MA 02115, USA; Thrombosis Research Group, Brigham and Women’s Hospital, 75 Francis Street, Boston, MA 02115, USA; Center for Cardiovascular Disease Prevention, Brigham and Women’s Hospital, Boston, MA, USA; Vascular Disease and Thrombosis Research Center, Rajaie Cardiovascular Institute, Tehran 1995614331, Iran; Cardiovascular Epidemiology Research Center, Rajaie Cardiovascular Institute, Tehran 1995614331, Iran; Clinical Trial Center, Rajaie Cardiovascular Institute, Tehran 1995614331, Iran

**Keywords:** Aspirin, Chewable, Coated, Air pollution, Randomized controlled trial, Atherosclerotic cardiovascular disease

## Abstract

**Aims:**

Although enteric-coated aspirin is widely used instead of chewable formulations in patients with atherosclerotic cardiovascular disease (ASCVD), its effects on cardiovascular and gastrointestinal outcomes remain uncertain. Separately, air pollution is a driver of cardiovascular risk, yet individual-level interventions have not been tested in outcomes trials.

**Methods and results:**

The Coated Or Chewable Aspirin in Patients with Established AThErosclerotic Disease and a Hybrid Strategy to Mitigate the Adverse Effects of AIR Pollution (COATED-AIR) is a randomized trial with a 2 × 2 factorial design planned to address these knowledge gaps. Patients with ASCVD receiving low-dose aspirin are considered for inclusion. Major exclusion criteria consist of being within 72 h of acute/unstable ASCVD or revascularization, triple antithrombotic therapy, active/recent bleeding, life expectancy <1 year, or other barriers or unwillingness for enrolment. Eligible consenting patients are randomized, in a double-blind fashion, to enteric-coated vs. chewable aspirin 81 mg daily and (via factorial randomization) to a hybrid strategy to mitigate the adverse effects of air pollution vs. usual care. The hybrid strategy includes (i) an educational flashcard, (ii) real-time air quality alerts via cellular text messages, and (iii) recommendations to (a) limit outdoor activity or (b) use a KN-95 facemask when outdoors, or (c) increase citrus fruit intake on days with elevated pollution levels. The primary efficacy outcome for the aspirin randomization is a composite of fatal/non-fatal ischaemic stroke, thrombotic/thromboembolic myocardial infarction, and acute limb ischaemia. For the AIR randomization, other forms of cardiovascular death are additionally included in the primary composite outcome. The median planned duration of follow-up is 2 years. Enrolment was completed on 28 January 2026. Clinical follow-up and blinded event adjudications are ongoing.

**Conclusion:**

COATED-AIR will determine whether aspirin coating affects its safety or effectiveness, and whether individual-level strategies can mitigate cardiovascular risks from air pollution in patients with ASCVD.

## Introduction

Aspirin is among the most widely prescribed antiplatelet agents for patients with atherosclerotic cardiovascular disease (ASCVD),^1–3^ with evidence of reducing ischaemic events compared with no treatment.^4^ However, its utility is tempered by antiplatelet-mediated systemic bleeding risk and direct gastrointestinal toxicity, including gastroduodenal ulcers and bleeding.^5,6^ Enteric-coated formulations, designed to bypass gastric absorption and reduce mucosal irritation, have been marketed as a solution, though their protective effect remains putative,^6–11^ with only a few, small clinical trials with short follow-up and inconclusive findings (*Figure 1*).^12–15^ In turn, enteric coating may alter pharmacokinetics, delay and reduce absorption, and potentially impair antithrombotic effectiveness, especially in the presence of food.^16–19^ Despite decades of clinical use, no outcomes trials have compared chewable vs. enteric-coated aspirin for thrombotic outcomes or gastrointestinal safety.^11^ While recent data suggest that P2Y_12_ inhibitors may have superior effectiveness, aspirin formulation was not considered in the individual clinical trials forming that evidence.^20–22^ Moreover, aspirin has better drug availability and affordability. Accordingly, understanding the differences between the major aspirin formulations remains a critical clinical question.

**Figure 1:**
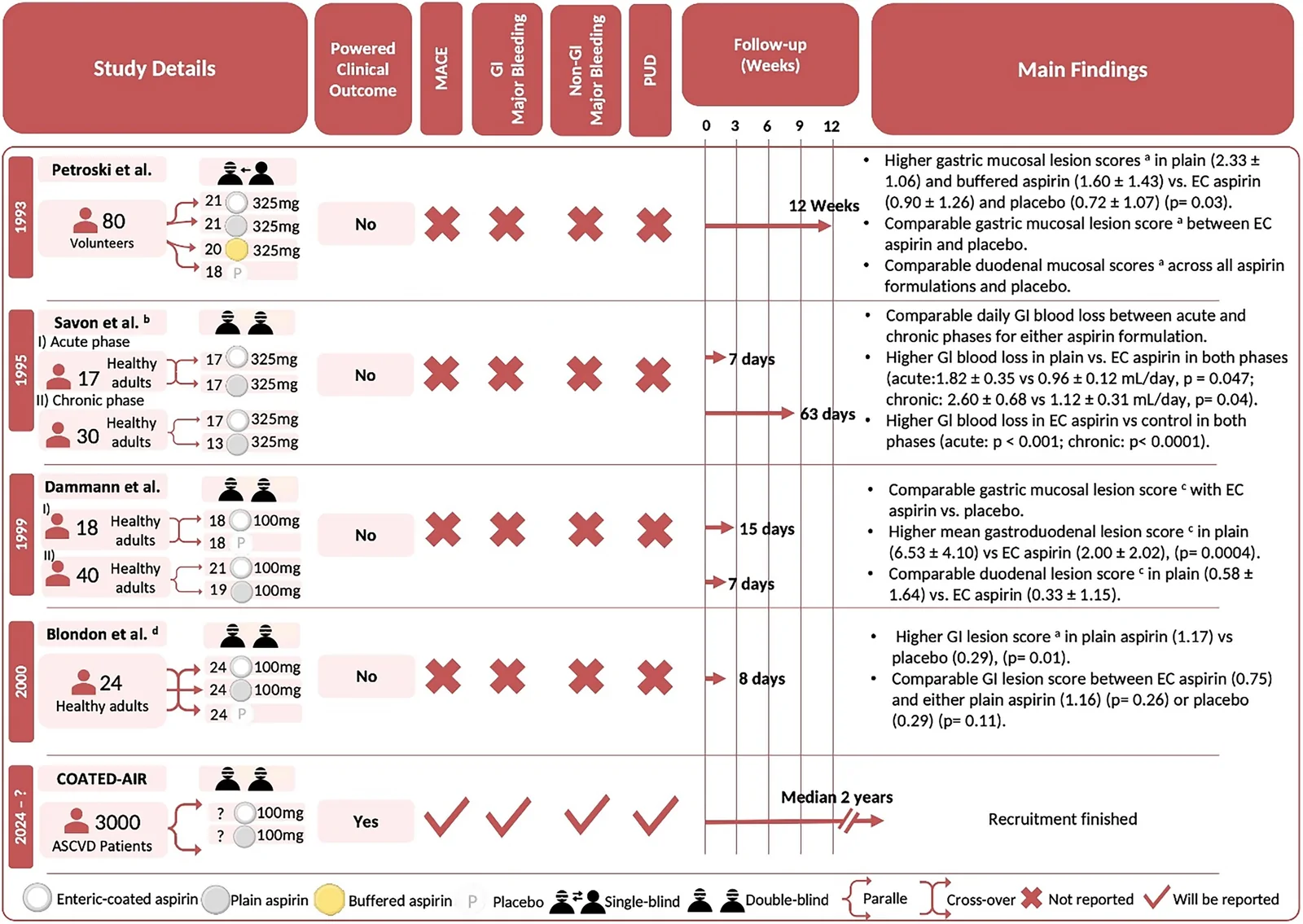
Randomized controlled trials evaluating safety outcomes of enteric-coated vs. plain aspirin.^12–15^  **^a^**Lanza score: **(0)** no lesions; **(1)** one submucosal haemorrhage or superficial ulcer; **(2 and 3)** 2 and ≥3 lesions; **(4)** extensive haemorrhage (with or without active bleeding) or invasive ulcers. **^b^**Pre-intervention gastrointestinal blood loss (stool sample) served as control, with each subject as their own control. Blinding not reported. **^c^**Modified Lanza score: **(0)** no identifiable lesions; **(1)** mucosal erythema; **(2–4)** ≥4/5–9/>9 petechiae; **(5–7)** ≥2/3–4/>4 erosions; **(8 and 9)** ≥1 ulcer. **^d^**Three-way crossover randomized trial. ASCVD, atherosclerotic cardiovascular disease; EC, enteric-coated; GI, gastrointestinal; MACE, major adverse cardiovascular event; PUD, peptic ulcer disease.

In addition to optimizing drug formulations, addressing environmental triggers represents another critical frontier in cardiovascular prevention. Air pollution is globally the fourth leading cause of mortality, responsible for 6.7 million annual deaths, nearly half from cardiovascular diseases.^23–25^ Pollutants such as particulate matter and nitrogen dioxide result in excessive inflammation and oxidative stress, thereby increasing the risk of myocardial infarction (MI), stroke, heart failure, and atrial fibrillation.^26–28^ While societal interventions such as emission regulations and traffic control remain resource-intensive and subject to policy changes, individual-level strategies^29–31^—including air quality alerts, facemasks, filtration systems, and antioxidant-rich diets—have gained traction. Yet, the impact of these interventions on cardiovascular outcomes remains largely untested in clinical outcomes trials (*Figure 2*).^32–35^ The Coated Or Chewable Aspirin in Patients with Established AThErosclerotic Disease and a Hybrid Strategy to Mitigate the Adverse Effects of AIR Pollution (COATED-AIR) randomized trial has been designed to address these two questions. This manuscript summarizes the key design features of COATED-AIR.

**Figure 2:**
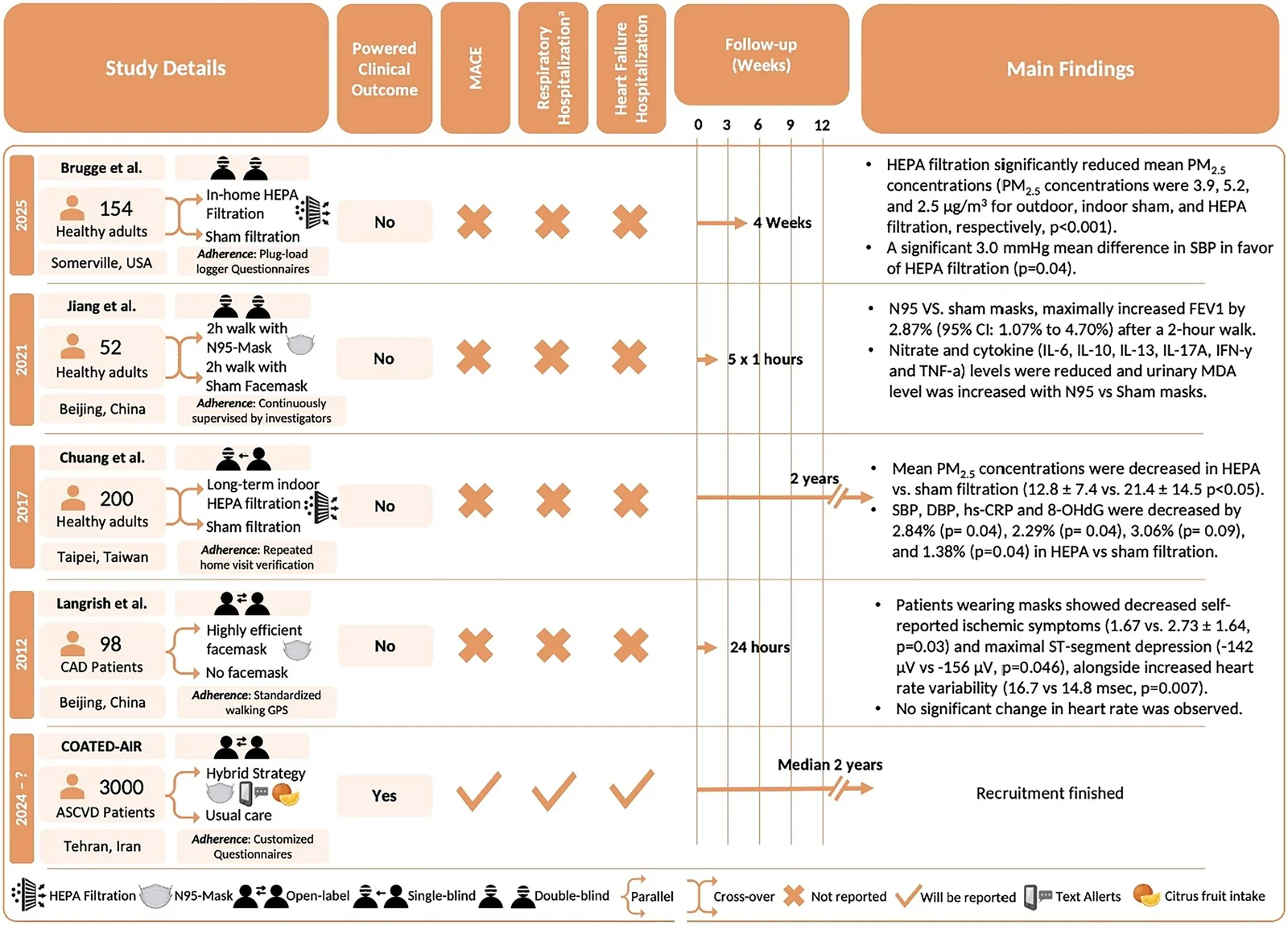
Randomized controlled trials evaluating individual-level air pollution mitigation interventions.^32–35 a^Respiratory-related events, including hospitalization for obstructive airway disease or pneumonia. ASCVD, atherosclerotic cardiovascular disease; CAD, coronary artery disease; DBP, diastolic blood pressure; FEV1, forced expiratory volume in 1 s; GPS, global positioning system; HEPA, high-efficiency particulate air; MACE, major adverse cardiac events; PM_2.5_, particulate matter with an aerodynamic diameter ≤2.5 µm; SBP, systolic blood pressure.

## Methods

### Overview

COATED-AIR is a randomized controlled trial with a 2 × 2 factorial design conducted across nine academic, community, and outpatient centres in Tehran, Iran—a city with major air pollution problems (*Figure 3*) (see Supplementary material online, Appendix).^26,36^ The first randomization involves double-blind assignment to chewable vs. enteric-coated low-dose (81 mg once daily) aspirin. The second randomization involves open-label assignment to a hybrid strategy to mitigate the cardiovascular adverse effects of air pollution vs. usual care. Tehran, the capital of Iran with nearly 10 million inhabitants, has experienced severe air pollution over the past decades, driven primarily by rapid growth in motor vehicles, industrial expansion, and accelerated urbanization. The city’s topography exacerbates pollution episodes, as the Alborz Mountains to the north and east restrict horizontal air movement and limit the dispersion of atmospheric pollutants. Mobile sources account for ∼70% of particulate matter emissions in the city. Air pollution is typically most severe during the cold season, when stable atmospheric conditions characterized by low wind speeds and temperature inversions inhibit pollutant dispersion.^37^ The Steering Committee (B.B., A.H.T., and P.S.) conceived the study and prepared an initial draft of the study protocol, which was subsequently reviewed and revised by all authors. An independent Data and Safety Monitoring Board (DSMB) inclusive of investigators not involved in the design or the conduct of COATED-AIR is in charge of oversight (see Supplementary material online, Appendix). The Institutional Review Board (IRB) of the Rajaie Cardiovascular Institute reviewed and approved the study protocol (IR.RHC.REC.1402.092), which was also approved by all other centres involved in patient recruitment. The trial is registered in clinicaltrials.gov (NCT06541691) and has been supported by an independent unrestricted research grant from Rajaie Cardiovascular Institute (grant number 1402092). The Actoverco company (Tehran, Iran) donated the aspirin formulations, in similar shape, colour, bottles, and boxes. None of these entities had any role in the design or execution of the trial, or the decision to prepare and submit the ensuing results.

**Figure 3:**
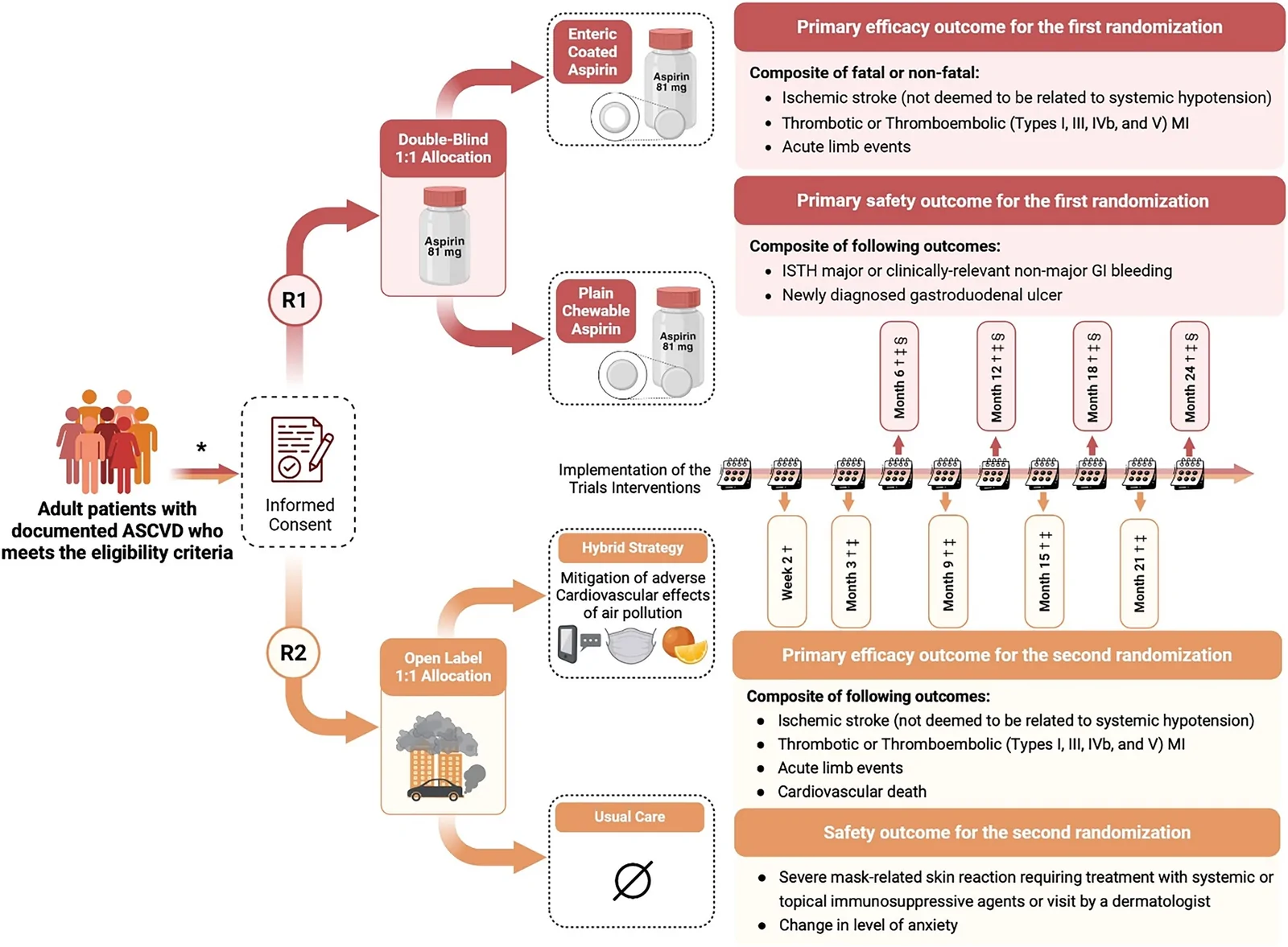
COATED-AIR trial scheme. Follow-ups will be continued beyond month 24 with similar regular follow-up visits until a primary efficacy endpoint occurs. * indicates the screening process (see the section *Screening and Randomization*) **†**Follow-ups will have assessment of study outcomes and adherence to the medication. **‡**Follow-ups will have assessment of adherence to the hybrid strategy (if randomized to hybrid strategy). **§**Follow-ups will have assessment of the quality of life (EQ-5D-5L), anxiety level (Generalized Anxiety Disorder-7), satisfaction with the hybrid strategy (if randomized to the hybrid strategy), and mask (if randomized to the hybrid strategy) and medication delivery. ASCVD, atherosclerotic cardiovascular disease; CV, cardiovascular; GI, gastrointestinal; ISTH, International Society on Thrombosis and Haemostasis; MI, myocardial infarction.

### Eligibility criteria

Patients aged >18 years in Tehran, Iran, with established ASCVD [including coronary artery disease (CAD), peripheral artery disease (PAD), carotid artery disease, ischaemic stroke not due to other causes such as atrial fibrillation or systemic hypoperfusion, or polyvascular atherosclerosis] who receive low-dose aspirin (75–100 mg once daily) and are planned to continue it are considered for inclusion (*Table 1*). Major exclusion criteria consist of being within 72 h of acute/unstable ASCVD events or revascularization, triple antithrombotic therapy, active bleeding at the time of screening or upper gastrointestinal or intracranial bleeding within 30 days prior to randomization or inherited or acquired severe coagulopathy, end-stage renal disease, or undergoing haemodialysis or peritoneal dialysis, aspirin sensitivity without successful desensitization,^38^ poor prognosis with an estimated life expectancy of <1 year according to the treating clinician, vascular disease exclusively from causes other than ASCVD, and allergy, or other barriers to the hybrid strategy. These barriers include inability or unwillingness to receive/read text messages/phone calls by personal mobile phone, or facial dysmorphia or unwillingness to use facemasks at the time of alerts.

**Table 1:** Trial eligibility criteria

Inclusion criteria	Exclusion criteria
Adult patients (aged ≥18 years) currently treated with aspirin and planned to continue the treatment with documented ASCVD defined as at least one of the following: Coronary artery disease (CAD): Previous or recent documented Type I myocardial infarction History of coronary revascularization (percutaneous coronary intervention or coronary artery bypass graft surgery) History of obstructive CAD (>50% stenosis) documented by coronary computed tomography^23^ or conventional angiography Peripheral artery disease (PAD): Previous or recent acute ischaemic limb event (>7 days prior) History of previous endovascular/surgical lower or upper extremities revascularization for an atherosclerotic cause History of lower extremity ulcer or amputation due to ASCVD Carotid artery disease: History of previous endovascular/surgical carotid artery revascularization for an atherosclerotic cause History of >50% carotid artery stenosis based on documented imaging tests (Duplex ultrasonography, CT angiography, magnetic resonance angiography, or conventional angiography) Ischaemic stroke: History of recent or previous documented ischaemic stroke not due to atrial fibrillation, endocarditis, or systemic hypoperfusion/hypotension, and being treated with low-dose aspirin Being inhabitant of Tehran province Willing to participate and able to provide written informed consent	Being within 72 h of acute/unstable ASCVD events (acute myocardial infarction, acute limb event, and acute ischaemic stroke), or within 72 h of revascularization Triple antithrombotic therapy Active bleeding History of upper GI bleeding within the past 30 days History of intracranial haemorrhage within the past 30 days End-stage kidney disease with estimated creatinine clearance <15 mL/min or undergoing haemodialysis or peritoneal dialysis Known aspirin sensitivity without prior successful desensitization Known comorbidities associated with poor prognosis (e.g. metastatic cancer) in conjunction with an estimated life expectancy of less than 1 year, according to the treating clinician Vascular disease known exclusively to be from causes other than atherosclerosis, including spontaneous coronary or peripheral dissection (e.g. fibromuscular dysplasia, segmental arterial mediolysis) or vasculitis (e.g. Takayasu arteritis, thromboangiitis obliterans, eosinophilic granulomatosis with polyangiitis) Inherited or acquired severe coagulopathies (e.g. haemophilia, decompensated liver cirrhosis) Not having aspirin as part of the planned durable treatment regimen Known allergy to KN-95 (or other masks), or citrus fruits Any facial dysmorphia that make the patient unable or unwilling to wear a facemask Necessity of unblinded facemask use for outdoor activities at the discretion of the treating clinician or based on patient preference Inability to receive/read text messages/phone calls by personal mobile phone (or that of a caregiver who lives with the patient and is willing to relay messages) Unwillingness to participate, such as hesitation to wear a mask, if randomized Any other unexpected/unwarranted conditions that make the participants unsuitable for recruitment or follow-up (e.g. illiteracy)

ASCVD, atherosclerotic cardiovascular disease; CAD, coronary artery disease; CT, computed tomography; GI, gastrointestinal; PAD, peripheral artery disease.

### Intervention(s) and comparator(s)

The first randomization involves double-blind assignment to enteric-coated vs. plain (chewable) low-dose (81 mg) aspirin once daily. For quality assurance purposes, samples will be systematically collected from each production batch of both enteric-coated and plain aspirin formulations. Each 16 000-bottle batch of enteric-coated and plain aspirin (8000 bottles per arm, 100 tablets per bottle) will be sampled. Thirty bottles of each type will be randomly selected. Six tablets per bottle will be tested for content, disintegration, and dissolution, and 10 tablets for physical traits (dimensions, coating thickness, and weight) to verify batch consistency and product quality. Both drug formulations are visually similar and packaged in identical bottles, ensuring that patients and clinicians remain blinded to treatment allocation.

The second intervention consists of a hybrid strategy to mitigate the adverse cardiovascular effects of air pollution, compared with usual care.^39^ The hybrid strategy includes the following components:

1. A one-page flashcard that outlines the adverse cardiovascular effects of air pollution and presents individual-level mitigation strategies.
2. Cell phone text messages to alert patients on days with air pollution levels exceeding the pre-specified thresholds (see Supplementary material online, Methods and Appendix) In such days, patients would consider the following recommendations:
  a. Avoid outdoor activities, especially exercise, to minimize time spent outdoors.
  b. Use KN-95 facemasks if they need to go outdoors for certain necessary activities.
  c. Consume citrus fruits (such as one medium orange, Supplementary material online, *Table S1*).

The hybrid strategy was developed by the Steering Committee based on literature review and pragmatic considerations to integrate exposure avoidance, physical filtration barriers (N95 respirators) when exposure is unavoidable, and dietary antioxidant supplementation (increased citrus fruit intake) to mitigate pollution-related oxidative stress and systemic inflammation. Fresh citrus fruits were selected as a practical dietary source of vitamin C (∼70–80 mg per serving) and other bioactive compounds with anti-inflammatory and antioxidant properties, and were preferred over supplemental formulations to capture the broader nutrient matrix of whole foods while enhancing accessibility. A dedicated clinical trial demonstrated the feasibility and lack of short-term perceived stress to participants from the hybrid strategy, compared with no intervention.^40^ The shared population, overlapping endpoints, and concurrent follow-up make the factorial design operationally efficient without compromising the scientific integrity of either comparison.

### Screening and randomization

Recruitment will take place through participating cardiovascular outpatient clinics and inpatient units at each recruiting centre. Potential candidates will be assessed by the site investigators who will assess the eligibility. For those who meet the eligibility criteria, the study will be discussed with the patient or their authorized healthcare proxy. Following the acquisition of written informed consent, eligible patients will proceed to randomization.

Randomization will follow a permuted block design, using block sizes of 8, 12, and 16, selected at random via a secure electronic web-based system. Eligible patients will be randomized in a factorial design, receiving assignments for both comparisons: (i) low-dose (81 mg once daily) enteric-coated vs. chewable (plain) aspirin formulation and (ii) a hybrid strategy vs. usual care. The study biostatistician defined the specifications for generating the randomization schedule. An independent biostatistician, not involved in trial operations, generated the schedule, including subject identification numbers, block numbers, randomization codes, and treatment allocations.

### Study outcomes

Although the primary outcome for both randomizations is largely similar, minor distinctions were strategically decided a priori (*Figure 4*). For the aspirin formulation randomization, the primary efficacy outcome encompasses thrombotic or thromboembolic events and is a composite of acute fatal or non-fatal thrombotic/thromboembolic MI (type 1, type 3, type 4b, and type 5 according to the Universal Definition of Myocardial Infarction^41^—as long as they were thrombotic/embolic in nature), ischaemic stroke, and acute ischaemic limb events. Key secondary outcomes include the individual components of the primary outcome, as well as all-cause death, cardiovascular death, and unplanned vascular hospitalization. The primary safety outcome for the aspirin randomization is a composite of major or clinically relevant non-major gastrointestinal bleeding (according to International Society on Thrombosis and Haemostasis criteria)^42^ and new symptomatic gastroduodenal ulcer.

**Figure 4:**
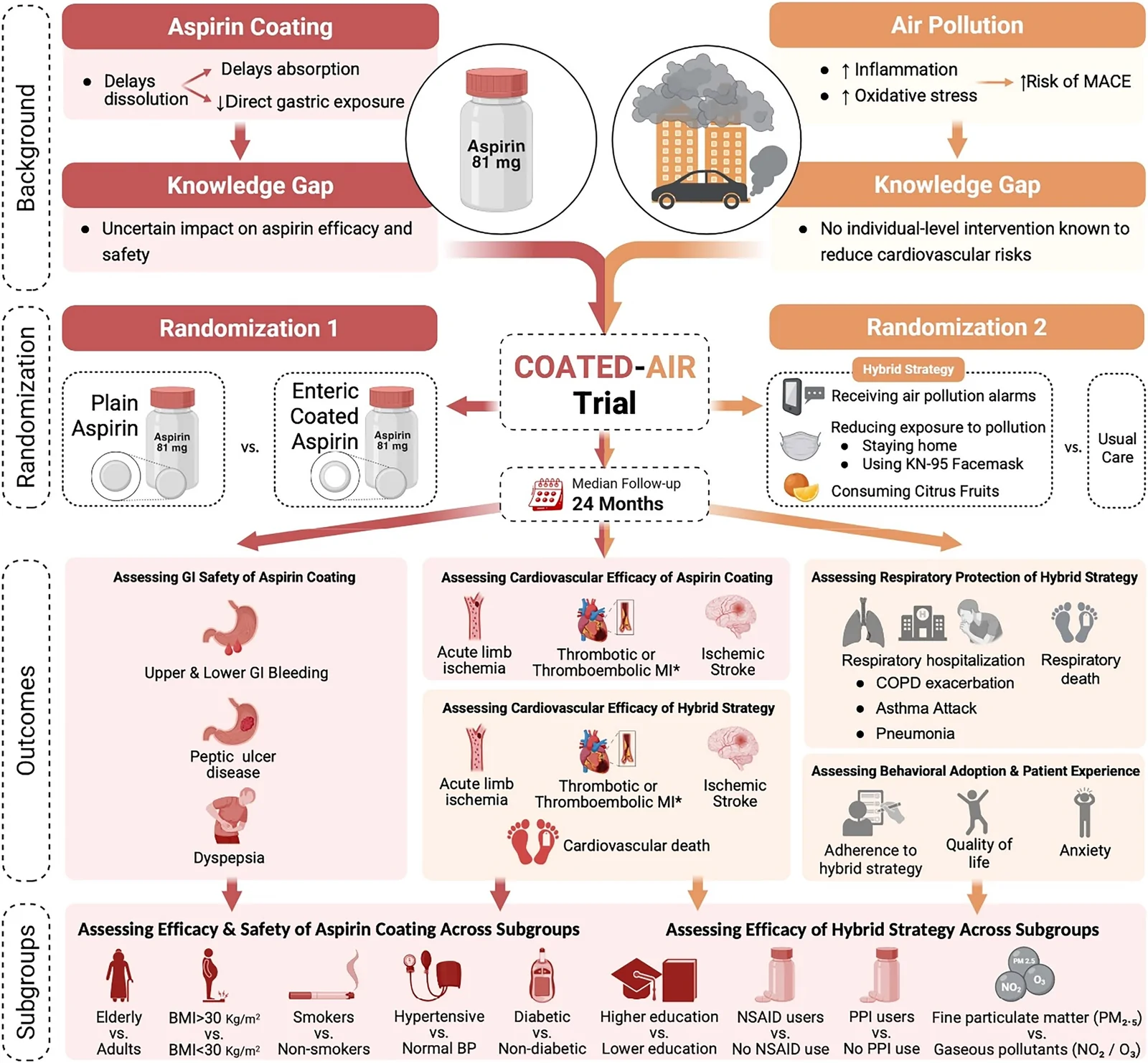
Key concepts for COATED-AIR design and outcomes. *Composed of Types I, III, IVb, and V myocardial infarction according to the Fourth Universal Definition of Myocardial Infarction, as long as they were thrombotic or thromboembolic in nature. BMI, body mass index; BP, blood pressure; GI, gastrointestinal; MACE, major adverse cardiovascular events; MI, myocardial infarction; NO_2_, nitrogen dioxide; NSAID, non-steroidal anti-inflammatory drug; O_3_, ozone; PPI, proton pump inhibitor.

For the hybrid strategy vs. usual care randomization, the primary efficacy outcome is a composite of acute non-fatal ischaemic stroke, thrombotic or thromboembolic MI, acute ischaemic limb events, or cardiovascular death. In addition to the individual components of the primary outcome, other key secondary outcomes include all-cause death, respiratory-related death, heart failure hospitalizations, and respiratory-related hospitalizations. While the primary efficacy outcomes for both randomizations are largely similar, non-thrombotic cardiovascular death was excluded from the aspirin primary outcome because non-thrombotic cardiovascular deaths (e.g. from heart failure or arrhythmia outside of acute coronary syndromes) are unlikely to be influenced by aspirin formulation, whereas they may plausibly be affected by air pollution exposure. The main safety outcomes for the second randomization include severe mask-related reactions requiring treatment with topical or systemic immunosuppressive agents or a visit by a dermatologist, and a change in the level of anxiety during follow-up periods, as assessed by the Generalized Anxiety Disorder-7 (GAD-7) questionnaire^43^ (see Supplementary material online, *Tables S2* and *S3*). The median planned duration of follow-up is 2 years. All outcomes will be adjudicated by a Clinical Events Committee whose members will be blinded to assigned treatments.

### Other data elements

COATED-AIR will also gather a rich set of additional data elements. These include demographic information, prior symptoms, comorbid conditions, revascularization history, baseline pharmacotherapy, and laboratory values. During follow-up visits, adherence to assigned interventions and patient satisfaction will be ascertained. In addition, quality of life will be assessed at baseline and during interval follow-up visits every 6 months using the EQ-5D-5L questionnaire. This scale measures the quality of life on a 5-component scale which includes mobility, self-care, usual activities, pain/discomfort, and anxiety/depression.^44,45^

Assessment of the air quality is an integral component of the second randomization. Daily assessment of the Air Quality Index^30^ from active air quality monitoring stations of Tehran will be used for the decision to send air pollution alerts (see Supplementary material online, Methods and Appendix). The daily level of different major air pollutants will also be collected. The geographic coordinates of the participants’ place of residence and the proximity of the nearest monitoring stations will be used to estimate individual daily exposure levels for future supplemental analyses.

### Data abstraction and quality control

Data abstraction will be performed by study physicians assigned to each study site under the auspices of the trial Coordinating Center. Multiple processes are implemented for quality control by the Coordinating Center. The online data entry system has built-in safeguards to avoid erroneous entry of implausible numbers (e.g. age 280 years). This is supplemented by unscheduled site visits, as well as manual review of a 5% sample of abstracted data.

### Sample size estimation

No prior randomized comparisons exist in secondary prevention populations with long-term follow-up data comparing enteric-coated vs. chewable aspirin. Therefore, we estimated the control arm event rate for composite thrombotic cardiovascular outcomes using data from other studies in Europe and North America.^46–50^ The estimated 24-month cumulative incidence of cardiovascular death and non-fatal MI based on prior studies was 12.1% [95% confidence interval (CI): 6.4%–17.9%]. Since these studies did not include acute limb events, we incorporated data from the Cardiovascular Outcomes for People Using Anticoagulation Strategies trial,^51^ yielding an adjusted estimate of 12.8% (95% CI: 6.7%–18.8%) for cardiovascular death, non-fatal MI, stroke, and acute limb events. We then adjusted for anticipated higher event rates at Iranian sites due to limited access to therapies and environmental contributors. A multicentre cohort of 1799 patients with acute coronary syndromes in 11 hospitals reported a 30% cumulative incidence for stroke/transient ischaemic attack, acute coronary syndromes, and death by 2-year follow-up.^52^ Accounting for endpoint differences, we estimated a cumulative incidence of 21.6% (95% CI: 18.5%–25.0%) at median 2-year follow-up for participants for the composite outcome. After iterative discussions, the COATED-AIR Steering Committee selected a control arm cumulative incidence higher than the Western estimates but lower than the Iranian cohort. As such, the lower bound of the 95% CI of the Iranian study was chosen: 18.5%.

Given the plausible difference in efficacy between enteric-coated and plain aspirin due to delayed absorption, and the absence of prior randomized trials comparing these formulations,^6,10^ the Steering Committee undertook an exploratory meta-analysis of aspirin vs. control in PAD populations, expected to comprise a substantial portion of COATED-AIR enrolees. This pooled analysis yielded a relative risk of 0.66, lower than the 0.81 rate ratio reported in the Antithrombotic Trialists' meta-analysis for secondary prevention,^4^ which excluded PAD trials. Considering the greater burden of atherosclerosis in our population and the mix of patients with CAD, stroke, and PAD, the Steering Committee estimated a relative risk reduction of 0.71 for aspirin vs. placebo. To conservatively account for preserved aspirin function across formulations, a hazard ratio (HR) of 0.77 for plain vs. coated aspirin was selected for sample size estimation. Assuming a two-sided alpha of 0.05, 80% power, and a control arm event rate of 18.5% over median follow-up of 24 months (with 4% loss to follow-up), 460 primary efficacy events will be required to detect a significant difference between chewable and coated aspirin. This translates to a total sample size of 2920 randomized participants.

No prior secondary prevention randomized trials evaluated strategies to mitigate cardiovascular risk from air pollution with a primary clinical endpoint. To approximate the control arm event rate, we extended the aspirin formulation estimate by incorporating cardiovascular deaths due to causes other than MI, stroke, and acute limb events. Based on the Prevention of Cardiovascular Events in Patients with Prior Heart Attack Using Ticagrelor Compared to Placebo on a Background of Aspirin-Thrombolysis in Myocardial Infarction 54 (PEGASUS-TIMI 54) trial,^49^ such causes account for ∼7.5% of composite cardiovascular outcomes, of which approximately half overlap with ischaemic stroke or limb events. Thus, an incremental 3.5%–4% relative increase was applied to our estimates for the second randomization, yielding an estimated cumulative incidence of 19.2% for the control group over a median follow-up period of 24 months.

With respect to treatment effect, no single component of the hybrid strategy—modification of outdoor activities, filtered masks, or dietary supplementation—has been tested in outcomes trials. However, epidemiologic studies have reported up to 25% hazard increases in cardiovascular events per 10 μg/m^3^ rise in PM_2.5_, with risks among individuals with established cardiovascular disease estimated to be 20% to 64% higher per 10 μg/m^3^ than in the general population.^27^ Conservatively, the Steering Committee considered a HR of 0.75 compared with usual care for the combined effect of the hybrid strategy. Assuming α = 0.05, 80% power, and 4% loss to follow-up, 380 primary events will be required, corresponding to a total sample size of 2732 participants for this randomization. Given the event-driven nature of these randomized comparisons, upon discussion with and the approval of the IRB, to maximize statistical power, it was decided to enrol all consenting participants of the aspirin randomization for this second randomization, as well.

### Statistical considerations

The primary analyses will be conducted in the modified intention-to-treat (mITT) cohort. For the first randomization (aspirin formulation), the mITT cohort will include all patients who were properly randomized (i.e. not meeting the exclusion criteria), did not withdraw consent for research data use, and received at least one dose of the assigned aspirin formulation. For the second randomization (hybrid strategy), inclusion in the mITT cohort will additionally require that the participant confirmed availability of a cell phone and ability to receive and read text messages during the trial welcome call. The safety cohort for both randomizations will be identical to the mITT cohort. Complementary analyses will include all randomized patients who did not withdraw consent for data use (intention-to-treat cohort), and per-protocol cohorts with ≥75% adherence to the assigned treatment.

A test of interaction for the respective primary outcomes of each intervention will be performed between the interventions. If a significant interaction (*P* < 0.05) is excluded, results for each randomization will be reported independently.

For each randomization, time-to-first-event analyses will be conducted for the pre-specified primary and key secondary clinical outcomes. Treatment effects will be summarized using a competing risks model, reporting HRs with 95% CIs. Kaplan–Meier estimates and Cox proportional hazards models will be used as a complementary analysis. Models will include indicators for the randomized intervention of interest. Proportional hazards assumptions will be evaluated using standard diagnostics. If proportionality is violated, treatment effects will also be summarized using restricted mean survival time differences over a predefined period. These will be estimated via flexible parametric or pseudo-value regression. Time-varying effects may also be assessed using Cox models with time-dependent coefficients for selective outcomes, such as quality of life or adherence to components of hybrid strategy for the second randomization.

Interim analyses will be planned after accrual of 50% (two-sided Type I error level α = 0.000063) and 75% (two-sided Type I error level α = 0.0027) of the target number of primary efficacy endpoint events for the aspirin randomization. Stopping rules for superiority for the primary efficacy outcome will be based on a modified Haybittle–Peto rule that necessitates a 4-SD difference at the initial 50% and a 3-SD difference at the subsequent interim analysis, also requiring consistency over 3 months for each. Similar procedures will be implemented for the hybrid strategy randomization. With minimal alpha expenditure at interim analyses, the traditional significance level of 0.05 will be used for the final analysis.

### Patient-reported outcomes (longitudinal)

Health-related quality of life will be assessed using the EQ-5D-5L at baseline and at 6-month interval follow-up visits. The primary EQ-5D-5L summary measure will be the utility index derived using an Iranian EQ-5D-5L value set.^53^ Longitudinal changes in EQ-5D-5L utility will be analysed using linear mixed-effects models with participant-specific random intercepts and fixed effects for time (modelled as a categorical factor for each 6-month visit), treatment group, and the time × treatment interaction. Estimated marginal means and between-group differences in change over time will be reported with 95% CIs. If linear mixed-effects model assumptions are violated, alternatives such as robust standard errors, outcome transformation, or generalized mixed-effects models will be considered. Sensitivity analyses under different missing-data assumptions may also be conducted. The five EQ-5D-5L dimensions (mobility, self-care, usual activities, pain/discomfort, and anxiety/depression) will be analysed as ordinal outcomes using cumulative logit mixed-effects models with the same fixed- and random-effects structure. Mixed-effects models were selected to account for repeated measurements and incomplete follow-up under a missing-at-random assumption.

Anxiety, as measured by the GAD-7 during follow-up, will be analysed using mixed-effects models appropriate to the scale (cumulative logit mixed-effects models when treated ordinally), with effects specified analogously to the EQ-5D-5L analyses.

### Hybrid-strategy adherence and satisfaction (intervention arm only)

Adherence to the components of the hybrid strategy and satisfaction with the strategy (collected only among participants assigned to the hybrid strategy) will be ascertained through follow-up calls, summarized descriptively and, where appropriate, analysed longitudinally within that group using mixed-effects models (linear mixed-effects models for continuous summary measures; ordinal mixed-effects models for Likert-type items). No between-group comparisons will be performed for these measures.

### Subgroup analyses

Pre-specified subgroup analyses will assess the consistency of randomized treatment effects using interaction terms, with *P* < 0.05 indicating a plausible potential interaction. Planned subgroups include age (≥65 vs. <65 years), sex, diabetes mellitus, current smoking status, use of a P2Y_12_ inhibitor at randomization, use of an anticoagulant at randomization, type of established ASCVD (CAD, PAD, ischaemic stroke, or >1 vascular bed), prior revascularization, history of peptic ulcer or gastrointestinal bleeding, baseline body weight, and baseline proton pump inhibitor use. For the air-pollution remediation efforts randomization, additional subgroups of interest include people with prior heart failure and with chronic lung disease.

### Enrolment progress

Enrolment started on 6 March 2024. Although the trial had no pre-specified goals for sex-based enrolment, a demographic analysis was performed after recruiting 2500 participants and revealed the inclusion of only 443 female participants and was discussed with the DSMB and the IRB. Neither body issued a mandate, largely due to prior knowledge of a higher prevalence of advanced ASCVD among male patients seeking care in Iran, conforming to clinical experience from several of the site investigators. However, the Steering Committee reviewed screening and enrolment patterns across sites. Of note, the Steering Committee, site Principal Investigators, and site physicians, all, included both women and men, and enrolment patterns were consistent across sites. Upon further review, we noted that although a large majority of screened participants (i.e. patients available for screening) had male sex, a greater proportion of female than male candidates were ineligible due to a lack of cell phone access or literacy to read text messages. Since both randomizations were event-driven, the Steering Committee proposed increasing the enrolment target to 3000 and to preferentially include females where possible for the remainder of trial enrolment, which the IRB and DSMB approved. Enrolment concluded on 28 January 2026, adding 199 additional female and 301 additional male individuals for a total of 3000 participants. Follow-up is ongoing.

## Discussion

COATED-AIR will be the first clinical outcomes trial to formally test two commonly encountered unresolved questions for patients with ASCVD: whether enteric coating impacts efficacy and safety of low-dose aspirin, and whether adverse cardiovascular effects of air pollution can be mitigated by a hybrid strategy. Despite decades of availability of both aspirin formulations, clinicians use them interchangeably or prefer one over another without clarity about their comparative effectiveness or safety. With the growing challenges of air pollution in many regions of the world, the second randomization to the hybrid strategy vs. usual care can test the effectiveness of a relatively easy and scalable intervention for potential mitigation of the adverse cardiovascular effects of air pollution. Data from COATED-AIR will also lend support to testing the effect of the randomized interventions on other outcomes such as respiratory-related hospitalizations, as well as epidemiological investigations, including those that test the association between air pollution indices and cardiovascular events in a clinical trial setting with adjudicated outcomes (*Figure 4*).

Aspirin continues to be widely used in patients with cardiovascular diseases.^54^ In addition to its ease of use and familiarity for perioperative non-cardiovascular procedures, aspirin has been linked in emerging data with potential reductions in incident cancer or the risk of metastatic disease. These tentative advantages of aspirin, as well as its low costs and global availability, make it likely that answering the questions about aspirin formulation remains very relevant in the contemporary era where many antiplatelet treatment strategies shift towards P2Y_12_ receptor inhibitors.^11,20^ In addition, the superior effectiveness signal for P2Y_12_ inhibitor in coronary disease is largely related to patients from East Asia.^21^ The assumed treatment effect for the aspirin randomization was informed by pharmacodynamic evidence of attenuated platelet inhibition with enteric coating and consensus in the Steering Committee members. Although COATED-AIR cannot exclude a smaller between-group difference, the investigative team considered the tested treatment effect to be clinically meaningful. Enteric coating has never been tested in an adequately powered outcomes trial, so its presumed gastrointestinal safety advantage over plain aspirin remains untested; a gap that COATED-AIR is designed to fill. If COATED-AIR indicates differential signals for effectiveness or safety of either aspirin formulation, the comparative effectiveness or the safety of the superior regimen vs. P2Y_12_ inhibitors should also be freshly revisited.

The randomization related to mitigation of adverse effects of air pollution is equally important. As we have summarized previously, we remain unaware of other completed or ongoing randomized trials powered for clinical endpoints that have sought to identify individual-patient-level strategies to mitigate the cardiovascular risk from air pollution.^40^

Emerging data indicate the utility long-term follow-up results for clinical trials after completion of the randomized interventions.^55^ Besides completing the main trial follow-up for a median of 24 months, the Steering Committee has also discussed a final 5-year long-term follow-up from study participants. All-cause death will be ascertained at 5-year follow-up while further follow-up for other outcomes will be contingent on securing ample resources to conduct such follow-up.

Despite the trial’s strengths and novelty, several limitations merit consideration. In the absence of prior cardiovascular outcomes trials to inform sample size estimation, the assumed event rates were based on Western secondary prevention data adjusted upward based on event rates from data from Iran. In the absence of prior cardiovascular outcomes trials, the treatment effects were based on mechanistic information and expert consensus, have not been validated in prior outcomes trials, and may prove optimistic. Nevertheless, COATED-AIR prioritized hard clinical endpoints, and while we remain blinded to breakdown of events per group, we are aware that events continue to accrue. Other individual completed randomized trialss in this space were >10-fold smaller than COATED-AIR and with short follow-up. Even if for unforeseen reasons the trial becomes underpowered compared with the intended design, it will still be a major step forward for our understanding of treatment trade-offs of coated vs. chewable aspirin and for the effect of the tested individual-level strategy to mitigate the adverse effects of air pollution. The second randomization is open-label, but outcomes will be blindly adjudicated. Finally, while randomization is limited to a single country, the pollution levels there are representative of similar air quality concerns in major cities worldwide. Given the scarcity of global data on individual-level air pollution mitigation strategies, COATED-AIR provides an important initial assessment of the effectiveness and safety of these therapies.

## Conclusions

In conclusion, COATED-AIR will provide robust evidence on the comparative effectiveness and safety of chewable vs. enteric-coated aspirin. Irrespective of the directionality of the results, findings will be practice-informing for patients who receive this treatment. Results from the hybrid strategy randomization will be equally important to understand whether there may be effective patient-level interventions to reduce the cardiovascular burden associated with air pollution.

## Supplementary Material

pvag071_Supplementary_Data

## Data Availability

Data from COATED-AIR trial are still being accrued. Upon completion, data can be requested through a reasonable research proposal submitted to the Steering Committee (Drs. Sadeghipour, Talasaz, and Bikdeli).
