# Supplementary material for: Coated or chewable aspirin and a hybrid air pollution mitigation strategy in patients with atherosclerotic disease: rationale and design of the COATED-AIR randomized trial
Source: Eur Heart J Cardiovasc Pharmacother. 2026 Sep 23;12(7):572–83. doi: 10.1093/ehjcvp/pvag071 (PMC13730551; doi:10.1093/ehjcvp/pvag071)
Supplement: Supplementary file 1 [file pvag071_supplementary_data.docx]

**Supplementary Appendix**

**Coated or chewable Aspirin and a hybrid strategy to mitigate the adverse effects of air pollution in patients with atherosclerotic disease: Rationale and Design of the COATED-AIR Randomized Clinical Trial**

Table of Contents

[Investigators and committees:	2](#_Toc231167116)

[Steering Committee:	2](#_Toc231167117)

[National Advisory Committee:	2](#_Toc231167118)

[International Advisory Committee:	2](#_Toc231167119)

[Pulmonary Advisory Committee:	3](#_Toc231167120)

[Trial statistician:	3](#_Toc231167121)

[Air Pollution Expert Advisor:	4](#_Toc231167122)

[Clinical Event Committee ^1^:	4](#_Toc231167123)

[Data Safety and Monitoring Board (DSMB):	5](#_Toc231167124)

[Trial Coordinating Centre (Rajaie Cardiovascular Institute):	5](#_Toc231167125)

[Recruiting Site Principal and Co-Investigators:	5](#_Toc231167126)

[Supplemental methods: Assessment of air quality and sending alerts	7](#_Toc231167127)

[Supplementary Table 1. Estimated vitamin C content of different citrus plants (based on the U.S. Department of Agriculture). This will be used as a practical surrogate for citrus fruit intake*	8](#_Toc231167128)

[Supplementary Table 2. Study outcomes	9](#_Toc231167129)

[Supplementary Table 3. Study outcomes operational definitions	10](#_Toc231167130)

# Investigators and committees:

## Steering Committee:

- **Behnood Bikdeli, MD, MS (Co-chair)**
- Heart and Vascular Institute, Brigham and Women’s Hospital, Mass General Brigham, Boston, MA, USA.
- Thrombosis Research Group, Brigham and Women’s Hospital, Boston, MA, USA.
- Harvard Medical School, Boston, MA, USA.
- YNHH/ Yale Center for Outcomes Research and Evaluation (CORE), New Haven, CT, USA.
- **Parham Sadeghipour, MD (Co-chair)**
- Vascular Disease and Thrombosis Research Center, Rajaie Cardiovascular Institute, Tehran, Iran
- Clinical Trial Center, Rajaie Cardiovascular Institute, Tehran, Iran
- **Azita H. Talasaz, PharmD (Co-chair)**
- Department of Pharmacy, New York-Presbyterian Hospital, Columbia University Medical Center, New York, NY, USA.
- Department of Pharmacotherapy and Outcomes Sciences, Virginia Commonwealth University, Richmond, VA, USA.

## National Advisory Committee:

- **Majid Maleki, MD**
- Vascular Disease and Thrombosis Research Center, Rajaie Cardiovascular Institute, Tehran, Iran
- **Feridoun Nouhi, MD**
- Vascular Disease and Thrombosis Research Center, Rajaie Cardiovascular Institute, Tehran, Iran

## International Advisory Committee:

- **Dominick J. Angiolillo, MD, PhD**
- Division of Cardiology, University of Florida College of Medicine, Jacksonville, FL, USA
- **Raffaele De Caterina, MD, PhD**
- University Cardiology Division, Pisa University Hospital, Pisa, Italy
- **Ajay J. Kirtane, MD, SM**
- Columbia University Irving Medical Center/NewYork-Presbyterian Hospital and the Cardiovascular Research Foundation
- **Harlan M. Krumholz, MD, SM**
- YNHH/ Yale Center for Outcomes Research and Evaluation (CORE), New Haven, CT, USA
- Section of Cardiovascular Medicine, Yale School of Medicine, New Haven, CT, USA
- Department of Health Policy and Management, Yale School of Public Health, New Haven, CT, USA
- **Loren Laine, MD**
- Section of Digestive Diseases, Yale School of Medicine, New Haven, CT, USA
- **Gregory Y.H. Lip, MD**
- Liverpool Centre for Cardiovascular Science at University of Liverpool, Liverpool John Moores University and Liverpool Heart and Chest Hospital, Liverpool, UK
- Department of Clinical Medicine, Aalborg University, Aalborg, Denmark
- **Renato D Lopes, MD, PhD**
- Department of Cardiology, Duke University, Durham, NC, USA
- **Roxana Mehran, MD**
- Icahn School of Medicine at Mount Sinai, NY, USA
- **Luis Ortega Paz, MD, PhD**
- Division of Cardiology, University of Florida College of Medicine, Jacksonville, FL, USA
- **Gregory Piazza MD, MS**
- Thrombosis Research Group, Brigham and Women’s Hospital, Harvard Medical School, Boston, MT, USA
- Division of Cardiovascular Medicine, Brigham and Women’s Hospital, Harvard Medical School, Boston, MT, USA
- **Sanjay Rajagopalan, MD, MBA**
- University Hospitals, Harrington Heart and Vascular Institute, Case Western Reserve University School of Medicine, Cleveland, OH, USA
- **Jayant K. Raikhelkar, MD**
- Division of Cardiology, Department of Medicine, Columbia University Irving Medical Center, NY
- **Paul M. Ridker, MD, MPH**
- Division of Cardiovascular Medicine, Brigham and Women's Hospital, and Harvard Medical School, Boston, MT, USA
- **Mehdi H. Shishehbor, DO, MPH, PhD**
- University Hospitals Harrington Heart and Vascular Institute, Cleveland, OH, USA
- **Philippe Gabriel Steg, MD**
- French Alliance for Cardiovascular Trials, Paris, France
- Université Paris-Cité, Paris, France
- INSERM Unité 1148, Paris, France
- Assistance Publique–Hôpitaux de Paris (AP-HP), Hôpital Bichat, Paris, France

## Pulmonary Advisory Committee:

- **David Jimenez, MD, PhD**
- Respiratory Department, Hospital Ramón y Cajal and Instituto Ramón y Cajal de Investigación Sanitaria IRYCIS, Madrid, Spain
- CIBER Enfermedades Respiratorias (CIBERES), Madrid, Spain
- Medicine Department, Universidad de Alcalá, Madrid, Spain
- **Luis Jara Palomares, MD, PhD**
- Medical Surgical Unit of Respiratory Diseases, Virgen del Rocio Hospital and Instituto de Biomedicina, Seville, Spain

## Trial statistician:

- **Hooman Bakhshandeh, MD, PhD**
- Vascular Disease and Thrombosis Research Center, Rajaie Cardiovascular Institute, Tehran, Iran
- Cardiovascular Epidemiology Research Center, Rajaie Cardiovascular Institute, Tehran, Iran
- Clinical Trial Center, Rajaie Cardiovascular, Institute, Tehran, Iran

## Air Pollution Expert Advisor:

- **Mohammad Sadegh Hassanvand, PhD**
- Department of Environmental Health Engineering, School of Public Health, Tehran University of Medical Sciences, Tehran, Iran
- Center for Air Pollution Research (CAPR), Institute for Environmental Research (IER), Tehran University of Medical Sciences, Tehran, Iran

## **Clinical Event Committee ^1^:**

- **Bahram Mohebbi, MD (Chair)**
- Vascular Diseases and Thrombosis Research Center, Rajaie Cardiovascular Medical and Research Institute, Tehran, Iran
- **Hessam Kakavand, PharmD (Coordinator)**
- Department of Clinical Pharmacy and Pharmacoeconomics, School of Pharmacy, Iran University of Medical Sciences, Tehran, Iran
- Cardiovascular Research Center, Rajaie Cardiovascular Institute, Tehran, Iran
- **Parham Sadeghipour, MD**
- Vascular Disease and Thrombosis Research Center, Rajaie Cardiovascular Institute, Tehran, Iran
- Clinical Trial Center, Rajaie Cardiovascular, Institute, Tehran, Iran
- **Behnood Bikdeli, MD, MS**
- Heart and Vascular Institute, Brigham and Women’s Hospital, Mass General Brigham, Boston, MA, USA.
- Thrombosis Research Group, Brigham and Women’s Hospital, Boston, MA, USA.
- Harvard Medical School, Boston, MA, USA.
- YNHH/ Yale Center for Outcomes Research and Evaluation (CORE), New Haven, CT, USA.
- **Azita H. Talasaz, PharmD**
- Department of Pharmacy, New York-Presbyterian Hospital, Columbia University Medical Center, New York, New York, USA
- Department of Pharmacotherapy and Outcomes Sciences, Virginia Commonwealth University, Richmond, Virginia, USA.
- **Seyed Hashem Sezavar, MD**
- Research center for prevention of Cardiovascular diseases, Institute of Endocrinology &Metabolism, Tehran, Iran
- **Maryam Mehrpooya, MD**
- Department of Cardiology, Imam Khomeini Hospital Complex, Tehran University of Medical Sciences, Tehran, Iran
- **Sheida Malekian, MD**
- Department of Internal Medicine, Shahid Modarres Hospital, Shahid Beheshti University of Medical Sciences, Tehran, Iran

## **Data Safety and Monitoring Board (DSMB):**

- **Babak Sharif-kashani, MD (Chair)**
- Lung Transplantation Research Center, Department of Cardiology, National Research Institute of Tuberculosis and Lung Diseases (NRITLD), Shahid Beheshti University of Medical Sciences, Tehran, Iran
- Tobacco Prevention and control Research center, National Research institute of Tuberculosis and Lung Diseases (NRITLD), Shahid Beheshti University of Medical Sciences, Tehran, Iran
- **Hassan Riahi, MD**
- Ghalb-e-Shoma Heart & Vascular Clinic, Tehran, Iran
- **Reza Ghanavati, MD**
- Ghalb-e-Shoma Heart & Vascular Clinic, Tehran, Iran
- **Samira Chaibakhsh, PhD (statistician)**
- Cardiovascular Epidemiology Research Center, Rajaie Cardiovascular Institute, Tehran, Iran

## Trial Coordinating Centre (Rajaie Cardiovascular Institute):

- **Trial Manager:** Sara Tayyebi, MS
- **Follow-up Centre:**
  - ***Study Physicians:*** Sepehr Jamalkhani, MD, Yasmin Mohtasham Kia, MD, Mahsa Nikkhoo, MD, Saghar Momeni, MD, MPH, Fatemeh Soltani, MS, Amir Abdi, MD
  - ***Staff:*** Maryam Zarrin Sadaf, Mohammad Gholipour,
- **Drug Support:** Naser Hadavand, PharmD, Ali Tavana, Hamid Abbas Nejad
- **Executive Group and drug delivery:** Navid Matlabi, Reza Khani, Morteza Tavana, Shokat Mahdavi, Fatemeh Sadeghi
- **IT Group:** Samira Kalaie Nia, PhD, Mahnaz Niknam, MS, Parisa Alidoust, PhD, Alireza Payamifard, Esmaeil Soumari, MS

## Recruiting Site Principal and Co-Investigators:

- **Rajaie Cardiovascular Institute, Tehran, Iran**

Parham Sadeghipour (Site Principal Investigator), MD, Ali Zahedmehr, MD, Bahram Mohebbi, MD, Razieh Omidvar, MD, Armin Elahifar, MD, Mohammad Javad Alemzadeh-Ansari, MD, Farshad Shakerian, MD, Ehsan Khalilipur, MD, Reza Kiani, MD, Mohsen Madani, MD, Amineh Safavirad, MD, Hossein Farshidi, MD, Hessam Kakavand, PharmD, Naser Hadavand, PharmD, Saeideh Mazloomzadeh, MD, PhD, Shiva Khaleghparast, PhD, Sepehr Jamalkhani, MD, Mahsa Nikkhoo, MD, Yasmin Mohtasham Kia, MD, Fatemeh Soltani, MS, Saghar Momeni, MD, MPH, Zahra Hashempour, MD, Amirhossein Poopak, MD, Erfan Kohansal, MD, Hamed Ghoshouni, MD, Mohammadreza Babaei, MD, Sara Montazeri Namin, MD, Fateme Yousefimoghaddam, MD, Amirali Soheili, MD, Zahra Ghaffarinejad, MD, Mahta Arbabi, MD

- **Tehran Heart Center, Tehran, Iran**

Maryam Aghakouchakzadeh (Site Principal Investigator), PharmD, Saeed Sadeghian, MD, Yaser Jenab, MD, Hassan Aghajani, MD, Mehdi Mehrani, MD, Mojtaba Salarifar, MD, Hamidreza Pourhosseini, MD, Hessam Khatami, MD, Yeganeh Karimi, MD, Soroush Nematollahi, MD, Mahshad Sabri, MD, Parisa Fallahtafti, MD

- **Sina Hospital, Tehran, Iran**

Mostafa Rouzitalab (Site Principal Investigator), MD, Abbas Soleimani, MD, Zahra Hashempour, MD, Mahsa Nikkhoo, MD, Sepehr Moghaddam, MD

- **Imam Khomeini Hospital, Tehran, Iran**

Maryam Mehrpooya (Site Principal Investigator), MD, Hamed Vahidi, MD, Mohammadreza Babaei, MD, Sepehr Moghaddam, MD, Mahshad Sabri, MD

- **Modarres Hospital, Tehran, Iran**

Saeed Alipour-Parsa (Site Principal Investigator), MD, Vahid Eslami, MD, Sheida Malekian, MD, Sarah Nourani, MD, Sara Montazeri Namin, MD

- **Hazrat Rasul Akram Educational, Research and Treatment Complex, Tehran, Iran**

Seyed Ali Banifatemeh (Site Principal Investigator), MD, Abdollah Amirfarhangi, MD, Mohsen Taghavi-Shavazi, MD, Zahra Hashempour, MD

- **Taleghani General Hospital, Tehran, Iran**

Mohammad Reza Beyranvand, MD, Mohammad Esmaeil Gheydari, MD, Mehdi Sheibani, MD, Sarah Nourani, MD

- **Private clinics, Tehran, Iran**

Seyed Hashem Sezavar, MD, Jalal Norouzi, MD, Hessam Khatami, MD

# Supplemental methods: Assessment of air quality and sending alerts

The air quality index ^2^ will be collected from all active air quality monitoring stations of Tehran province by an assigned research assistant at 7:00 A.M on a daily basis. Such data will be acquired from the online Tehran air quality map available from Tehran Air Quality Control Company and the Iranian Department of Environment. If the median AQI among all stations was higher than a prespecified cutoff, an air pollution alert will be sent to the patients in the hybrid strategy arm at 8:00 A.M. This timeline was adopted due to pragmatic reasons (i.e., timely delivery of alerts to allow patients staying at home). The prespecified cutoff was considered as >120 for September 21^st^ through March 20^th^ (i.e., fall/winter), and >100 for March 21^st^ to the end of September 20^th^ (i.e., spring/summer).

| **Supplementary Table 1.** Estimated vitamin C content of different citrus plants (based on the U.S. Department of Agriculture). This will be used as a practical surrogate for citrus fruit intake* | | | |
| --- | --- | --- | --- |
| **Fruit** | **Portion** | **Vitamin C content** | **Equivalent RDA** |
| Orange | One medium size | 70 mg | One medium size |
| Tangerine | One medium size | 23.5 mg | 3 medium size |
| Lime | One medium size (Juiced) | 19.5 mg | 3 (large) / 4 (medium) Juiced |
| Lemon | One small size (Juiced) | 31 mg | 2 (large) / 3 (medium) Juiced |
| Sweet lemon | One medium size | 50 mg | 1.5 medium size |
| Bitter orange | One medium size | 45-90 mg | 1–1.5 medium size |
| RDA, recommended dietary allowance.  * The steering committee decided not to target vitamin C, per se, as part of the intervention, and rather preferred only natural sources, and in that, only citrus fruit. While patients in both groups have no restrictions of taking natural or supplemental vitamin C, by virtue of being randomized, it is expected that such use is balanced between the groups and unlikely to have clear spikes on days of excess air pollution, in the absence of randomized encouragement. | | | |

| **Supplementary Table 2.** Study outcomes | |
| --- | --- |
| **First randomization: Aspirin formulation** | |
| **Efficacy** | **Safety** |
| - **Primary efficacy outcome:**  1. Composite of fatal or nonfatal ischemic stroke (not deemed to be related to systemic hypotension), thrombotic or thromboembolic myocardial infarction (type 1, 3, 4b or 5), and acute ischemic limb events  - **Secondary efficacy outcomes:**  1. Ischemic stroke (not deemed to be related to systemic hypotension) 2. thrombotic or thromboembolic myocardial infarction (type 1, 3, type 4b or 5) 3. Acute ischemic limb event 4. All-cause death 5. Cardiovascular death 6. Unplanned vascular hospitalization  - **Exploratory efficacy outcomes:**  1. Stent thrombosis 2. Unplanned revascularization 3. Major amputation 4. Heart failure hospitalization 5. Venous thromboembolism 6. Atrial fibrillation | - **Primary Safety outcome:**  1. Composite of ISTH major or clinically-relevant non-major GI bleeding and new symptomatic diagnosed gastroduodenal ulcer  - **Secondary safety outcomes:**  1. Upper GI bleeding 2. ISTH clinically relevant non-major GI bleeding 3. Any ISTH major bleeding 4. Intracranial hemorrhage 5. Any ISTH clinically-relevant non-major bleeding 6. New symptomatic diagnosed gastroduodenal ulcer 7. Dyspepsia leading to drug discontinuation or switch to open-label enteric-coated aspirin |
| **Second randomization: Air pollution** | |
| **Efficacy** | **Safety** |
| - **Primary efficacy outcome:**  1. Composite of non-fatal ischemic stroke (not deemed to be related to systolic hypotension), thrombotic or thromboembolic myocardial infarction (type 1, 3, or type 4b or 5), acute limb events, or cardiovascular death  - **Secondary efficacy outcomes:**  1. Ischemic stroke (not deemed to be related to systemic hypotension) 2. thrombotic or thromboembolic myocardial infarction (type 1, 3, or type 4b or 5) 3. Acute ischemic limb event 4. Cardiovascular death 5. All-cause death 6. Respiratory-related death 7. Heart failure hospitalization 8. Respiratory-related hospitalization  - **Exploratory outcomes:**  1. Adherence to all components of the hybrid strategy 2. Satisfaction of patients with the hybrid strategy 3. Change in the health-related quality of life 4. Venous thromboembolism 5. New onset atrial fibrillation | - **Safety outcome:**  1. Severe mask-related skin reaction requiring treatment with systemic or topical immunosuppressive agents or a visit by a dermatologist 2. Change in the level of anxiety |
| ISTH, International Society on Thrombosis and Haemostasis | |

| **Supplementary Table 3.** Study outcomes operational definitions | |
| --- | --- |
| **Outcomes** | **Definitions** |
| **Efficacy outcomes** | |
| ***Acute ischemic stroke*** | An acute episode of neurological dysfunction caused by focal cerebral, spinal, or retinal infarction, not attributed to systemic hypotension^3^ |
| ***Acute thrombotic or thromboembolic myocardial infarction*** | Any episode of type 1, 3, 4b, or 5 myocardial infarction according to the fourth universal definition of myocardial infarction^4^ that were deemed to be thrombotic or thromboembolic in nature. |
| ***Acute ischemic limb events*** | An acute, limb-threatening impairment of arterial blood flow to an extremity, documented by either objective hemodynamic evaluation or vascular imaging, and requiring urgent vascular intervention, including pharmacologic treatment, surgical revascularization, percutaneous angioplasty, stent placement, or limb amputation, within 30 days of symptom onset ^5^ and were deemed thrombotic or thromboembolic in nature. |
| ***Cardiovascular death*** | Death resulting from cardiovascular causes, including acute myocardial infarction, sudden cardiac, including unwitnessed death, heart failure, stroke, cerebrovascular hemorrhage, or other cardiovascular causes such as pulmonary embolism, acute aortic syndromes, and arrhythmia^6^. |
| ***All-cause death*** | Death due to any cause (cardiovascular, non-cardiovascular, or undetermined)^6^ |
| ***Respiratory-related death*** | Any death caused by chronic obstructive pulmonary disease exacerbation, asthma attack, or other forms of obstructive or restrictive lung disease, pneumonia, hypoxemic and/or hypercapnic respiratory failure as the main feature of critical illness leading to death in the absence of any more likely cause. While there is overlap, death due to pulmonary embolism will be adjudicated as cardiovascular |
| ***Unplanned vascular hospitalization*** | Inpatient admission to a hospital specifically due to acute events involving the vascular system, including acute myocardial infarction, ischemic or hemorrhagic stroke, acute limb ischemia, and other vascular emergencies necessitating urgent medical care and hospitalization^5^ |
| ***Stent thrombosis*** | Any event of type 4b myocardial infarction according to the fourth universal definition of myocardial infarction^4^ |
| ***Unplanned revascularization*** | Any urgent or emergent percutaneous or surgical revascularization due to events such as acute limb ischemia or acute coronary syndrome, to restore blood flow to affected areas and prevent further ischemic damage^5^ |
| ***Major amputation*** | Any ankle disarticulation, transtibial/below-knee amputation, or transfemoral/above-knee amputation^7^ |
| ***Heart failure hospitalization*** | Admission for ≥24 h (or change of calendar date if admission and discharge time are lacking) with a primary diagnosis of heart failure, and the subject has objective evidence of a new or aggravated pre-existing right and/or left-sided heart failure meeting the criteria for heart failure hospitalization at the clinician’s discretion^8^ |
| ***Venous thromboembolism*** | Any episode of acute pulmonary embolism (confirmed by CT angiography, ventilation/perfusion scan, invasive pulmonary angiography or echocardiography (thrombus visualized in the main pulmonary artery), and/or deep venous thrombosis (diagnosed in the upper/lower extremity, the inferior vena cava, deep splanchnic veins based on ultrasonography, or contrast-enhanced vascular imaging). ^9^ |
| ***Atrial fibrillation*** | New diagnosis of atrial fibrillation/flutter as confirmed by a 12-lead ECG, telemetry tracing, or other conventional modalities.^9,10^ |
| ***Respiratory-related hospitalization*** | Hospital stays > 24 hours for chronic obstructive pulmonary disease or asthma exacerbation, or pneumonia |
| ***Change in the health-related quality of life*** | Change in the patients’ quality of life throughout the study follow-up, assessed by EuroQol-5 Dimensions-5 Levels. The scale measures the quality of life on a 5-component scale including mobility, self-care, usual activities, pain/discomfort, and anxiety/depression^11,12^ |
| **Safety outcomes** | |
| ***New symptomatic diagnosed gastroduodenal ulcer*** | Gastroduodenal ulcer manifested by gastrointestinal symptoms that persist for > 3 days, with confirmation of ulcer by endoscopy. |
| ***Upper GI bleeding*** | Bleeding originating from sites in the esophagus, stomach, or duodenum with manifestations including hematemesis (vomiting of red blood or coffee-grounds material), melena (black, tarry stool), or hematochezia (passage of red or maroon material per rectum) |
| ***ISTH clinically relevant non-major GI bleeding*** | Any episode of GI bleeding that meets the ISTH definitions for clinically-relevant non-major bleeding |
| ***Any ISTH major bleeding*** | Any episode of bleeding, either GI or non-GI, that meets the ISTH definitions for major bleeding ( Bleeding that is fatal, occurs in a critical organ, or causes a hemoglobin drop of 2 g/dL or more, or requires transfusion of 2 or more blood units).^13^ |
| ***Intracranial hemorrhage*** | The abnormal bleeding within the confines of the skull, involving either the brain tissue (intracerebral hemorrhage) or the spaces between the layers that cover the brain (subarachnoid or subdural hemorrhage) |
| ***Any ISTH clinically-relevant non-major bleeding*** | Any episode of bleeding that meets the ISTH definitions for clinically-relevant non-major bleeding.^14^ |
| ***Dyspepsia*** | New or worsening epigastric pain, discomfort, postprandial fullness, or early satiety, present for at least for several days (at the discretion of treating clinician) and associated with initiating aspirin therapy, and not improved by H2receptor antagonists or proton pump inhibitors, or resulting in endoscopy, or associated with bleeding, nausea, vomiting, or markedly reduced appetite. |
| ***Severe mask-related skin reaction*** | Adverse skin reactions occurring due to the use of facial masks, which mandate dermatological consultation or the use of topical/systemic corticosteroids or other immunosuppressive agents. |
| ***Change in the level of anxiety*** | Change in the patients’ anxiety level throughout the study follow-up, assessed by Generalised Anxiety Disorder Assessment 7-item questionnaire. |
| GI, gastrointestinal; ISTH, International Society on Thrombosis and Haemostasis | |

**References**

1.	Mustafić H, Jabre P, Caussin C, et al. Main Air Pollutants and Myocardial Infarction: A Systematic Review and Meta-analysis. JAMA 2012;307(7):713-721, doi:10.1001/jama.2012.126

2.	Ren J, Liang J, Wang J, et al. Vascular benefits of vitamin C supplementation against fine particulate air pollution in healthy adults: A double-blind randomised crossover trial. Ecotoxicol Environ Saf 2022;241(113735, doi:10.1016/j.ecoenv.2022.113735

3.	Bozkurt B, Hershberger RE, Butler J, et al. 2021 ACC/AHA Key Data Elements and Definitions for Heart Failure: A Report of the American College of Cardiology/American Heart Association Task Force on Clinical Data Standards (Writing Committee to Develop Clinical Data Standards for Heart Failure). Circulation: Cardiovascular Quality and Outcomes 2021;14(4):e000102, doi:doi:10.1161/HCQ.0000000000000102

4.	Thygesen K, Alpert JS, Jaffe AS, et al. Fourth Universal Definition of Myocardial Infarction (2018). Circulation 2018;138(20):e618-e651, doi:doi:10.1161/CIR.0000000000000617

5.	Eikelboom JW, Connolly SJ, Bosch J, et al. Rivaroxaban with or without Aspirin in Stable Cardiovascular Disease. New England Journal of Medicine 2017;377(14):1319-1330, doi:doi:10.1056/NEJMoa1709118

6.	Garcia-Garcia HM, McFadden EP, Farb A, et al. Standardized End Point Definitions for Coronary Intervention Trials: The Academic Research Consortium-2 Consensus Document. Circulation 2018;137(24):2635-2650, doi:10.1161/circulationaha.117.029289

7.	Long CA, Mulder H, Fowkes FGR, et al. Incidence and Factors Associated With Major Amputation in Patients With Peripheral Artery Disease: Insights From the EUCLID Trial. Circ Cardiovasc Qual Outcomes 2020;13(7):e006399, doi:10.1161/circoutcomes.119.006399

8.	Abraham WT, Psotka MA, Fiuzat M, et al. Standardized Definitions for Evaluation of Heart Failure Therapies: Scientific Expert Panel From the Heart Failure Collaboratory and Academic Research Consortium. JACC Heart Fail 2020;8(12):961-972, doi:10.1016/j.jchf.2020.10.002

9.	Dehmer GJ, Badhwar V, Bermudez EA, et al. 2020 AHA/ACC Key Data Elements and Definitions for Coronary Revascularization: A Report of the American College of Cardiology/American Heart Association Task Force on Clinical Data Standards (Writing Committee to Develop Clinical Data Standards for Coronary Revascularization). Circ Cardiovasc Qual Outcomes 2020;13(4):e000059, doi:10.1161/hcq.0000000000000059

10.	Hindricks G, Potpara T, Dagres N, et al. 2020 ESC Guidelines for the diagnosis and management of atrial fibrillation developed in collaboration with the European Association for Cardio-Thoracic Surgery (EACTS): The Task Force for the diagnosis and management of atrial fibrillation of the European Society of Cardiology (ESC) Developed with the special contribution of the European Heart Rhythm Association (EHRA) of the ESC. Eur Heart J 2021;42(5):373-498, doi:10.1093/eurheartj/ehaa612

11.	EuroQol--a new facility for the measurement of health-related quality of life. Health Policy 1990;16(3):199-208, doi:10.1016/0168-8510(90)90421-9

12.	Emrani Z, Akbari Sari A, Zeraati H, et al. Health-related quality of life measured using the EQ-5D-5 L: population norms for the capital of Iran. Health Qual Life Outcomes 2020;18(1):108, doi:10.1186/s12955-020-01365-5

13.	Schulman S, Kearon C. Definition of major bleeding in clinical investigations of antihemostatic medicinal products in non-surgical patients. J Thromb Haemost 2005;3(4):692-4, doi:10.1111/j.1538-7836.2005.01204.x

14.	Kaatz S, Ahmad D, Spyropoulos AC, et al. Definition of clinically relevant non-major bleeding in studies of anticoagulants in atrial fibrillation and venous thromboembolic disease in non-surgical patients: communication from the SSC of the ISTH. J Thromb Haemost 2015;13(11):2119-26, doi:10.1111/jth.13140
